# Toxicity and Estrogenic Endocrine Disrupting Activity of Phthalates and Their Mixtures

**DOI:** 10.3390/ijerph110303156

**Published:** 2014-03-14

**Authors:** Xueping Chen, Shisan Xu, Tianfeng Tan, Sin Ting Lee, Shuk Han Cheng, Fred Wang Fat Lee, Steven Jing Liang Xu, Kin Chung Ho

**Affiliations:** 1 Vitargent (International) Biotechnology Limited, Unit 516, 5/F. Biotech Centre 2, No. 11 Science Park West Avenue, Hong Kong Science Park, Shatin, Hong Kong; E-Mails: cindy.tan@vitargent.com (T.T.); Kathy.lee@vitargent.com (S.T.L.); 2 State Key Laboratory in Marine Pollution, Department of Biology and Chemistry, City University of Hong Kong, 83 Tat Chee Avenue, Kowloon, Hong Kong; E-Mails: ssanyecao@gmail.com (S.X.); bhcheng@cityu.edu.hk (S.H.C.); 3 School of Science and Technology, Open University of Hong Kong, 30 Good Shepherd Street, Homantin, Kowloon, Hong Kong; E-Mails: wflee@ouhk.edu.hk (F.W.F.L.); sjlxu@ouhk.edu.hk (S.J.L.X.); kcho@ouhk.edu.hk (K.C.H.)

**Keywords:** phthalate, toxicity, estrogenic endocrine disruptor, estrogenic activity, enhanced-estrogenic activity, mixture effects

## Abstract

Phthalates, widely used in flexible plastics and consumer products, have become ubiquitous contaminants worldwide. This study evaluated the acute toxicity and estrogenic endocrine disrupting activity of butyl benzyl phthalate (BBP), di(*n*-butyl) phthalate (DBP), bis(2-ethylhexyl) phthalate (DEHP), diisodecyl phthalate (DIDP), diisononyl phthalate (DINP), di-*n*-octyl phthalate (DNOP) and their mixtures. Using a 72 h zebrafish embryo toxicity test, the LC_50_ values of BBP, DBP and a mixture of the six phthalates were found to be 0.72, 0.63 and 0.50 ppm, respectively. The other four phthalates did not cause more than 50% exposed embryo mortality even at their highest soluble concentrations. The typical toxicity symptoms caused by phthalates were death, tail curvature, necrosis, cardio edema and no touch response. Using an estrogen-responsive *ChgH*-*EGFP* transgenic medaka (*Oryzias melastigma*) eleutheroembryos based 24 h test, BBP demonstrated estrogenic activity, DBP, DEHP, DINP and the mixture of the six phthalates exhibited enhanced-estrogenic activity and DIDP and DNOP showed no enhanced- or anti-estrogenic activity. These findings highlighted the developmental toxicity of BBP and DBP, and the estrogenic endocrine disrupting activity of BBP, DBP, DEHP and DINP on intact organisms, indicating that the widespread use of these phthalates may cause potential health risks to human beings.

## 1. Introduction

Phthalates are diesters of phthalic acids that are commonly used as plasticizer to increase the flexibility, pliability and elasticity of plastics, and also widely used in cosmetics, personal care products, food packaging and medical products [1] With annual production of about 6.0 million tons, phthalates have been detected in water [2], air [3], sediments [4], soil [5], food [6,7], human blood plasma [1], breast milk [8,9], urine [10] and so on. The large production volume and wide application has made the presence of phthalates almost ubiquitous. Phthalates were revealed to be obesogens in recent studies [11,12]. The phthalates dimethyl phthalate (DMP), diethyl phthalate (DEP), DBP, BBP, DEHP and DNOP are classified by the United States Environmental Protection Agency (USEPA) as priority environmental pollutants [13]. 

Mainly due to their endocrine disrupting potency, which causes adverse health effects, phthalates that used to be regarded as safe are now of wide concern. Some phthalates were reported to have the potential to cause decreased testicular weight and seminiferous tubular atrophy, increased DNA damage in men’s sperm, premature breast development in girls, shortened pregnancy and decreased anogenital distance in newborn male babies (summarized by Schecter *et al*. [7]). These reproductive defects most likely result from the estrogen disrupting activity of certain phthalates.

Though studies have been carried out to evaluate the estrogenic endocrine disrupting activity of phthalates, and some phthalates such as DEP, DBP, BBP and DEHP have been reported to possess estrogenic activity *in vitro* [14,15,16,17,18,19,20], the toxicity and estrogenic endocrine disrupting potency of some phthalates and phthalate mixtures in *in vivo* systems are still not clear. 

The present study used zebrafish (*Danio rerio*) embryos and estrogen-responsive *ChgH*-*EGFP* transgenic medaka (*Oryzias melastigma*) eleutheroembryos to evaluate the developmental toxicity and estrogenic endocrine disrupting potency of BBP, DBP, DEHP, DIDP, DINP, DNOP and their mixtures. These six phthalates are of concern for their potential migration from food containers into the food and are regulated for toys and childcare products by the European Union [21] and some other countries.

## 2. Materials and Methods

### 2.1. Chemicals

**Figure 1:**
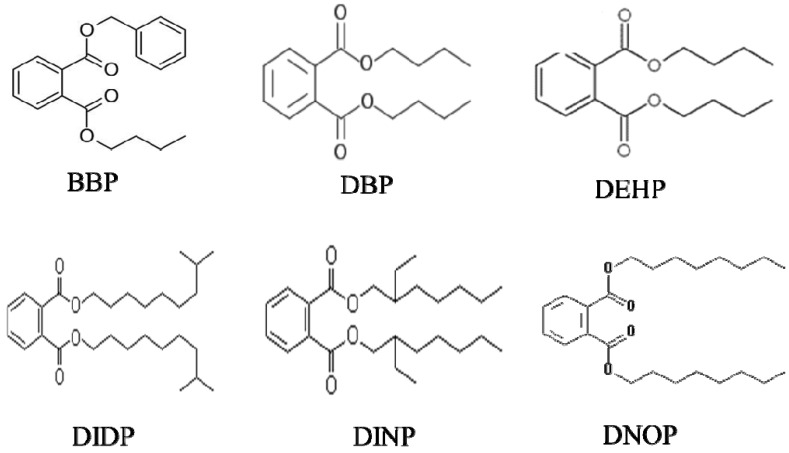
Chemical structure of phthalates.

The phthalates used in this study are Butyl benzyl phthalate (BBP, CAS# 85-68-7) from Aldrich, Di(n-butyl) phthalate (DBP, CAS# 84-74-2), Di(2-ethylhexyl) phthalate (DEHP, CAS# 117-81-7) and Diisodecyl phthalate (DIDP, CAS# 26761-40-0) from CHEM Service, Diisononyl phthalate (DINP, CAS# 28553-12-0) and Diisononyl phthalate (DNOP, CAS# 117-84-0) from Wako. The chemical structures of these phthalates are shown in Figure 1. As phthalates are oily substances and cannot be properly dissolved in water, so methanol was used as vehicle to increase their solubility in water. The final concentration of vehicle in the exposure solutions was 0.1%. 17-β-Estradiol (E2) standard was bought from the China National Standard Material Centre (Beijing, China). E2 stock solutions were prepared using methanol. 

### 2.2. Zebrafish (Danio rerio) Maintenance and Zebrafish Embryo Toxicity Test

Zebrafish AB strain was purchased from the Zebrafish International Resource Center (ZIRC) at the University of Oregon (Eugene, OR, USA). Adult zebrafish was maintained and fish embryos were collected as previously described [22]. Healthy embryos of 4–128 cell stages were used for the tests. Static exposure was carried out using 24-well plates. Each well contained one embryo, and each concentration was tested on 20 embryos. Phthalates and phthalate mixture (containing equal volumes of each phthalate at the stated concentration) were tested at 500.00, 100.00, 50.00, 10.00, 1.50, 0.60, 0.30, 0.06 and 0.01 ppm. Solvent (0.1% methanol) and blank control were also tested. After 72 h exposure, embryos were observed under a stereomicroscope (Leica MZ10F, Leica, Hong Kong, China) and photographed using a CCD camera (Leica DFC 310 FX) connected to the stereomicroscope. The mortality rate of embryos exposed to each concentration was scored at the end of the test. The lethal concentration 50 (LC_50_) was calculated based on the mortality-dose response curve.

### 2.3. Transgenic Medaka (Oryzias melastigma) Maintenance

A stable transgenic *O. melastigma* strain containing the promoter of the estrogen-dependent liver-specific *choriogenin H* (*ChgH*) gene regulating enhanced green fluorescence protein (*EGFP*) gene coding region (*ChgH*-*EGFP*) was developed [23,24]. Transgenic medaka was cultured at 2.5 ppt water at 26 ± 1 °C with a constant 12 h-light/12 h-dark photoperiod and fed with both brine shrimp (*Artemia salina*) and commercial flake food. Transgenic eleutheroembryos, the stage of hatched fish that still rely on the yolk for energy supply and have not started external feeding, were used for this study. 

### 2.4. Estrogenic Endocrine Disrupting Activity Test

Based on the zebrafish embryo toxicity test results and solubility of phthalates in water, preliminary tests were performed to find out if individual phthalates and the phthalate mixture possessed estrogenic endocrine disrupting activity (including estrogenic activity, enhanced-estrogenic activity and anti-estrogenic activity). The concentrations used for preliminary test were 1.50, 0.60, 0.30, 0.06 and 0.01 ppm for BBP and DBP, 50.00, 10.00, 1.50, 0.60, 0.30, 0.06 and 0.01 ppm or DEHP, DIDP, DINP and DNOP, and 1.5, 0.06 and 0.01 ppm for the phthalate mixture. For both estrogenic activity and enhanced-/anti-estrogenic activity pre-testing, about eight *ChgH*-*EGFP* transgenic medaka (*O. melastigma*) eleutheroembryos were exposed to each concentration of phthalate or phthalate mixture for 24 hrs and these were then observed under a stereomicroscope (Leica MZ10F) equipped with a UV excitation light source and GFP filter set (excitation filter BP470/40 nm). If green fluorescence signal (GFP) was observed in the liver of exposed eleutheroembryos that means that the phthalate or phthalate mixture possesses estrogenic activity; if no GFP was observed in the liver of exposed eleutheroembryos that means that phthalate or phthalate mixture possess no estrogenic activity and their enhanced-/anti-estrogenic activity were then pre-tested. To test enhanced-/anti-estrogenic activity testing, E2 (e.g., 1.00 or 2.50 ppb) and E2 (e.g., 1.00 or 2.50 ppb) together with phthalate or phthalate mixture were tested and their induced GFP signal were observed and compared. 

For easier comparison between different phthalates, one concentration (except for the phthalate mixture, which was tested at 0.15 and 1.50 ppm) that seems to induce, if there was any, an obvious signal was selected for formal testing. Formal testing exposures were performed using 24-well plates. Each concentration was tested in triplicate and each replicate contained eight eleutheroembryos. Methanol and water (0.1%) were included as solvent and blank control, respectively. E2 solutions of 1.00, 2.00, 5.00 and 10.00 ppb were tested as positive reference. After 24 h exposure, the liver of eleutheroembryos were imaged using a CCD camera (Leica DFC 310 FX) connected to the fluorescence stereomicroscope (Leica MZ10F, Leia, Hong Kong, China). The grey value (brightness) of each eleutheroembryo’s liver GFP signal intensity, represented as the relative fluorescence unit (RFU), were measured using image analysis software MetaMorph (Molecular Devices, Hong Kong, China). Data analysis were performed according to the ISO 20281 guidance [25] using Excel (Microsoft, Redmond, WA, USA). Student’s *t*-test (*p* < 0.05) was performed to define the stastically significant enhanced-/anti-estrogenic activity of phthalate mixtures. 

## 3. Results

### 3.1. Acute Toxicity of Phthalates and Their Mixtures

**Figure 2:**
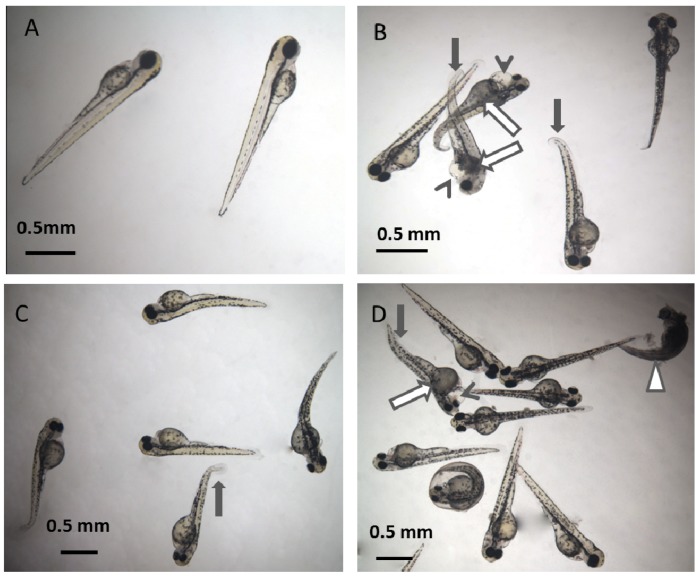
Representative photos of zebrafish (*Danio rerio*) embryos exposed to phthalates and phthalate mixture. (**A**): control; (**B**): 0.50 ppm BBP; (**C**): 1.50 ppm DBP; (**D**): mixture containing 0.50 ppm of BBP, DBP, DEHP, DIDP, DIDP and DNOP, respectively. Representative toxicity symptoms caused by phthalates includes death (empty triangle), tail curvature (solid arrow), necrosis (empty arrow), cardio edema (arrowhead) and no touch response.

The acute toxicity of phthalates and their mixtures to zebrafish embryos were analyzed in this study. The lethal concentration 50 (LC_50_) of phthalates and their mixture were calculated basing on their mortality-dose response curve. Of the six phthalates, DBP is the most toxic, with LC_50_ of 0.63 ppm, followed by BBP, with LC_50_ of 0.72 ppm. Though DEHP, DIDP, DINP and DNOP caused toxicity symptoms at high concentrations, they did not resulted to more than half zebrafish embryos death even at their highest soluble concentrations (the highest soluble concentration is close to 500.00 ppm for DEHP and <500.00 ppm for DIDP, DINP and DNOP) and their LC_50_ was not calculated. When the 6 phthalates were volume equally mixed together, additive toxicity effect was observed and its LC_50_ was calculated to be 0.50 ppm (containing 0.50 ppm of each of the six phthalates), more toxic than any of the 6 phthalates alone. The typical toxicity symptoms caused by phthalate included death, tail curvature, cardio edema, necrosis and no touch response (see Figure 2). 

### 3.2. Estrogenic Activity of Phthalates

**Figure 3:**
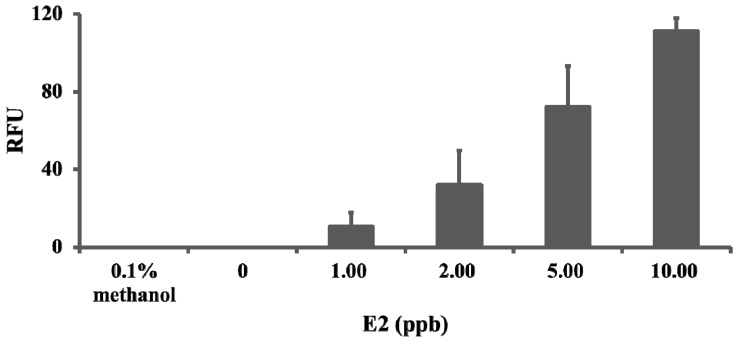
Dose-dependent induction of GFP in transgenic medaka (*O. melastigma*) eleutheroembryos by 17β-estradiol (E2). Transgenic eleutheroembryos were exposed to varying concentrations of E2 for 24 hrs and the induced GFP signal intensity (RFU) was measured (see Material and Methods for detail). No GFP was induced in solvent (0.1% methanol) and blank (0) controls.

Note: Data are mean ± standard error of the mean.

**Figure 4:**
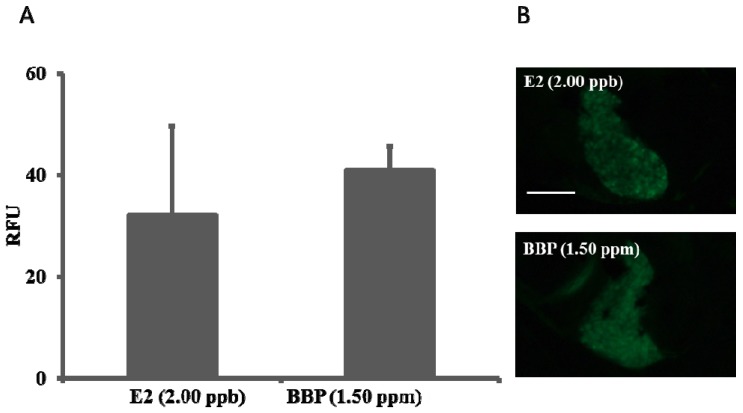
Estrogenic activity of phthalate BBP. (**A**): BBP (1.50 ppm) induced GFP signal intensity (RFU) compared to that of E2 (2.00 ppb), data are mean ± standard error of the mean; (**B**): Representative photos of the livers of transgenic medaka (*O. melastigma*) eleutheroembryos after 24-h exposure to E2 (2.00 ppb) and BBP (1.50 ppm). Scale bar equals to 100 µm.

Basing on the zebrafish embryo acute toxicity test results the estrogenic activity of the 6 phthalates were screened and further tested using estrogen-responsive *ChgH-EGFP* transgenic medaka *O. melastigma* eleutheroembryos. Results showed that no GFP expression was induced in the liver of transgenic eleutheroembryos exposed to solvent and blank controls, and the GFP expression level, represented as relative fluorescence unit (RFU), induced by E2 was obviously dose-dependent (Figure 3). Of the 6 phthalates tested, only BBP (1.50 ppm) induced GFP expression and the intensity of which was close to that of 2.00 ppb E2 (Figure 4). This means BBP possess estrogenic activity and is an estrogenic chemical. 

### 3.3. Enhanced-/Anti-estrogenic Activity of Phthalates

Phthalates DBP, DEHP, DIDP, DINP and DNOP that showed no estrogenic activity were processed for enhanced-/anti-estrogenic activity screening and testing. Transgenic eleutheroembryos were exposed to E2 (2.50 ppb) and E2 (2.50 ppb) together with phthalate for 24 h, and their induced green fluorescence intensity (RFU) were measured and analyzed

**Figure 5:**
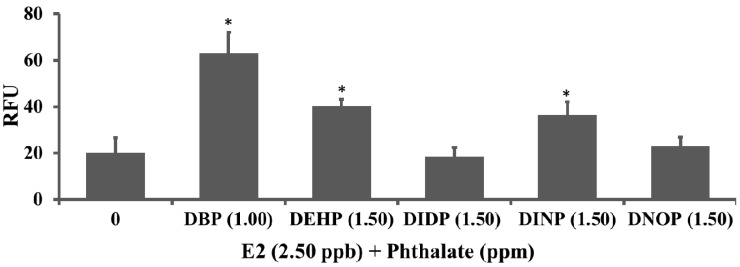
Enhanced-estrogenic activity of phthalates. DBP (1.00 ppm), DEHP (1.50 ppm) and DINP (1.50 ppm) significantly increased, and DIDP (1.5 ppm) and DNOP (1.5 ppm) did not statistically increase or decrease, the GFP signal intensity (RFU) of the livers of transgenic medaka (*O. melastigma*) eleutheroembryos induced by E2 (2.5 ppb).

Notes: Data are mean ± standard error of the mean; * denotes *p* < 0.05.

Results showed that 1.00 ppm DBP, 1.50 ppm DEHP or DINP significantly increased the green fluorescence intensity (RFU) induced by E2, indicating the enhanced-estrogenic activity of the three phthalates (Figure 5). DIDP and DNOP did not significantly affect (increase or decrease) the GFP signal intensity (RFU) induced by E2 (Figure 5), meaning these two phthalates are not estrogenic endocrine disrupting compounds.

### 3.4. Estrogen Disrupting Activity of Phthalate Mixtures

**Figure 6:**
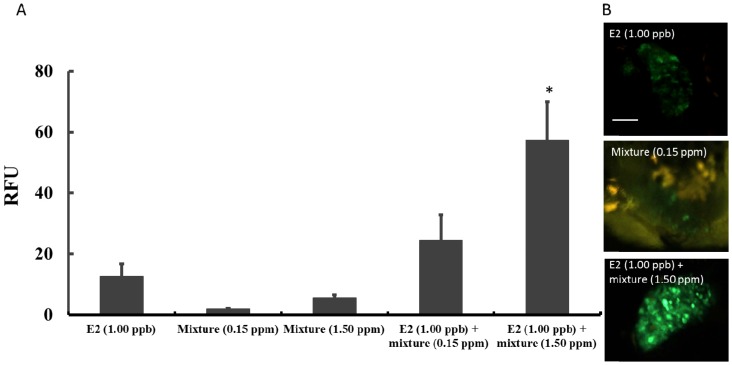
Enhanced-estrogenic activity of phthalate mixtures. (**A**): The GFP signal intensity (RFU) induced by E2 (1.00 ppb), mixtures containing 0.15 and 1.50 ppm of BBP, DBP, DEHP, DIDP, DINP and DNOP respectively, and E2 together with mixtures of 0.15 and 1.50 ppm respectively. (**B**): Representative images of the livers of transgenic medaka (*O. melastigma*) eleutheroembryos after 24-h exposure to E2 (1.00 ppb), phthalate mixture (0.15 ppm) and E2 (1.00 ppb) + phthalate mixture (1.50 ppm) respectively.

Notes: Data are mean ± standard error of the mean; * denotes *p* < 0.05; Scale bar equals to 100 µm.

Further tests were performed to find out whether mixtures of the six phthalates have estrogenic or enhanced-/anti-estrogenic activity. Phthalates were mixed at the stated concentrations and tested. Figure 6A shows that phthalate mixtures at 0.15 and 1.50 ppm induced very week GFP signals (RFU), demonstrating the weak estrogenic activity of the phthalate mixtures. However, when co-exposed with E2 (1 ppb), phthalate mixture at 1.50 ppm mixture, but not 0.15 ppm, significantly increased the transgenic *O. melastigma* eleutheroembryos’ liver GFP signal intensity (RFU). Representative photos of the livers of transgenic eleutheroembryos after 24 h exposure to E2 (1.00 ppb), phthalate mixture (0.15 ppm) and E2 (1.00 ppb) + phthalate mixture (1.50 ppm) respectively, are also presented (Figure 6B).

## 4. Discussion and Conclusions

Phthalates are ubiquitous in our daily life and may reach high concentrations in certain products and human populations. According to European Food Safety Authority regulation [26,27,28,29,30] regulation, the specific migration limits in food simulants are 30 mg/kg (*i.e.*, 30 ppm), 0.3 mg/kg (*i.e.*, 0.3 ppm) and 1.5 mg/kg (*i.e.*, 1.5 ppm) for BBP, DBP and DEHP respectively and the compositional limits in food contact materials are 0.1% (*i.e.*, 100 ppm) for BBP, DEHP, DIDP, DINP and DNOP and 0.05% (*i.e.*, 50 ppm) for DBP respectively. Based on these legislations, Danish scientists measured the contents of BBP, DBP, DEHP, DIDP and DINP in 100 officially collected food-contact material samples and found total 23 non-compliant samples.

Out of the 23 non-compliant samples, six samples contained 0.5%–5% DBP, 17 samples contained 0.05%–50% DEHP, five samples contained 25-50% DIDP and five samples contained 5%–50% DINP [31]. In foods, BBP, DBP and DEHP were reported to be detected at a rate of 95.2%, 90.5%, and 90.5% in edible vegetable oil in USA [32] and DBP concentrations reached up to 20.76 ± 6.60 ppb and 66.78 ± 7.60 ppb in cow fresh milk and milk powder respectively [8]. In humans, DBP and DEHP were reported to be the most commonly detected phthalates in breast milk with concentrations ranging from low ppb level in Germany [9] up to dozens ppb level (57.78 ± 35.42 ppb DBP) in China [8]; and in central Taiwan the total phthalate concentration in urine samples were reported to be 0.40 ppm (geometric mean) for 2-years-olds and 5-years-olds and 0.20 ppm (geometric mean) for pregnant women [10]. 

Using a 72-h zebrafish embryo acute toxicity test, this study demonstrated that BBP, DBP and a mixture of BBP, DBP, DEHP, DIDP, DINP and DNOP (*i.e.*, containing 0.50 ppm of each phthalate) possessed high toxicity, with LC_50_ values of 0.72 ppm, 0.63 ppm and 0.50 ppm, respectively, and induced severe developmental toxicity in live embryos. The high developmental toxicity of BBP and DBP revealed in present study and their reported comparatively high concentrations in the human body [8,10] should raise concerns about their potential health risks to human beings, especially vulnerable groups such as pregnant women and children. 

Besides acute toxicity, this study also evaluated the *in vivo* estrogenic and enhanced-estrogenic activity of the six phthalates and their mixtures using *ChgH*-*EGFP* transgenic *Oryzias melastigma* eleutheroembryos. Of the six tested phthalates, BBP exhibited detectable estrogenic activity, DBP, DEHP and DINP demonstrated enhanced-estrogenic activity, and DIDP and DNOP showed no enhanced- or anti-estrogenic activity. When the six phthalates were mixed at the same concentration, weak estrogenic activity and significant enhanced-estrogenic activity were detected at a 1.50 ppm level. Comparing the data of Figure 5 and Figure 6, no additive estrogenic activity or enhanced-estrogenic activity of DBP, DEHP and DINP was observed for mixtures at the tested concentrations. Of course, detailed phthalate mixture effects await further investigation.

The estrogenic endocrine disrupting activity test results of present study are generally consistent with that of previous reports, especially *in vivo* studies. To be more specific, BBP, though it caused no estrogenic effect in one ovariectomized Sprague-Dawley rat based an *in vivo* assay using uterine wet weight and vaginal cell cornification as endpoints [20], it has repeatedly demonstrated estrogenic activity in the present and several previous *in vitro* studies [15,16,18,20]. DBP that reportedly exhibited estrogenic activity in some *in vitro* tests [15,16,33], did not induce detectable estrogenic activity signal in the present and other *in vitro* and *in vivo* studies [20,34]. DEHP, like DBP, showed estrogenic activity in some *in vitro* and *in vivo* systems [19,35], but not in present and other *in vitro* and *in vivo* systems [20,33,36,37]. DIDP consistently showed no estrogenic activity in present and previous *in vitro* and *in vivo* assays [15,20]. DINP, except for very weak estrogenic activity exhibited in an *in vitro* assay [36] showed no estrogenic activity in the present and previous *in vitro* and *in vivo* tests [15,20]. DNOP, like DIDP, also consistently showed no estrogenic activity in present and previous *in vitro* and *in vivo* studies [20,38]. To conclude, phthalates that have been reported to possess estrogenic activity were identified to be either estrogenic (BBP), or enhanced-estrogenic (DBP, DEHP and DNOP) chemicals, and phthalates that consistently were reported to possess no estrogenic activity were further proved to be neither estrogenic nor enhanced-/anti-estrogenic chemicals in the present study.

The present study also reported the estrogenic and enhanced-estrogenic activity of phthalate mixtures. The results are in some sense consistent with that of a previous ER-luc transfected MVLN cell line study, which showed the mixture of BBP, DBP, dioctyl phthalate (DOP), DIDP, DINP, DEHP, bis(2-ethylhexyl) adipate (DEHA), 4-*tert*-octylphenol (tOP), 4-chloro-3-methylphenol (CMP), 2,4-dichlorophenol (2,5-DCP), 2-phenylphenol (2-PP) and resorcinol significantly induced the transactivation of ER in an additive manner [15]. Compared with a single chemical analysis, few studies have been carried out to investigate the enhanced-/anti-estrogenic activity of mixtures, which may be explained by several reasons including, but not limited to, the unavailability of proper testing methods, the complexity of chemical interactions in mixtures, and the difficulty of mimicking real situations. 

In conclusion, using two fish models, this study demonstrated the comparatively high toxicity of BBP and DBP, confirmed the estrogenic activity of BBP, and revealed the enhanced-estrogenic activity of DBP, DEHP, DINP and mixture of BBP, DBP, DEHP, DIDO, DINO and DNOP. This study also demonstrated the potential of using genetically modified fish eleutheroembryos for efficient estrogenic endocrine disruptors testing. 
